# Intelligent Evaluation Method of Human Cervical Vertebra Rehabilitation Based on Computer Vision

**DOI:** 10.3390/s23083825

**Published:** 2023-04-08

**Authors:** Qiwei Du, Heting Bai, Zhongpan Zhu

**Affiliations:** 1 College of Electronic and Information Engineering, Tongji University, Shanghai 201804, China; 2 Frontiers Science Center for Intelligent Autonomous Systems, Shanghai 201210, China

**Keywords:** cervical vertebra rehabilitation, computer vision, cervical spondylosis, evaluation method, scoring system

## Abstract

With the changes in human work and lifestyle, the incidence of cervical spondylosis is increasing substantially, especially for adolescents. Cervical spine exercises are an important means to prevent and rehabilitate cervical spine diseases, but no mature unmanned evaluating and monitoring system for cervical spine rehabilitation training has been proposed. Patients often lack the guidance of a physician and are at risk of injury during the exercise process. In this paper, we first propose a cervical spine exercise assessment method based on a multi-task computer vision algorithm, which can replace physicians to guide patients to perform rehabilitation exercises and evaluations. The model based on the Mediapipe framework is set up to construct a face mesh and extract features to calculate the head pose angles in 3-DOF (three degrees of freedom). Then, the sequential angular velocity in 3-DOF is calculated based on the angle data acquired by the computer vision algorithm mentioned above. After that, the cervical vertebra rehabilitation evaluation system and index parameters are analyzed by data acquisition and experimental analysis of cervical vertebra exercises. A privacy encryption algorithm combining YOLOv5 and mosaic noise mixing with head posture information is proposed to protect the privacy of the patient’s face. The results show that our algorithm has good repeatability and can effectively reflect the health status of the patient’s cervical spine.

## 1. Introduction

Cervical spondylosis is ranked as one of the most common pain problems, especially in Asia. According to [1], China had a pain incidence ratio of over 30% prior to the year 2016. Nearly 150 million people in China suffer from cervical spondylosis, and figures showed a gradual trend towards a younger average age. Of all the groups that develop cervical spondylosis, the adolescent group that suffers from it deserves our special attention [2,3,4,5,6]. According to medical research, active exercise of the neck, shoulder, and back muscles can enhance muscle strength, reinforce the normal physiological curvature of the cervical spine, increase the stability of the biomechanical structure of the cervical spine, and promote blood and lymphatic circulation, which is beneficial to the prevention and health recovery of cervical spondylosis [7]. However, due to the diverse types of cervical spondylosis and complex pathogenesis, such as cervical osteoarthritis, cervical disc disease, cervical stenosis, vertebral fracture and malignancies, the rehabilitation effects of the intensity and frequency of cervical spine rehabilitation exercises on different types of cervical spine diseases are not clear. This is limited by key issues such as the absence of cervical spine rehabilitation measurement methods and the lack of big data on the rehabilitation process. Moreover, in traditional medical programs, in the face of cervical spondylosis, medical experts are often required to manually guide patients through rehabilitation training, manually assess the recovery status of the patient’s cervical spine based on experience, and make adjustments to the rehabilitation training program accordingly, in order to enable patients with cervical spondylosis to fully recover. However, traditional medical rehabilitation training programs require professional guidance at all times, with low unmanned levels and high labor costs. To this end, this paper proposes a cervical vertebra rehabilitation evaluation method based on a computer vision algorithm using artificial intelligence technology. In addition, a cervical spine exercise training dataset is constructed for algorithm validation to provide artificial intelligence technology support for long-term tracking of patient health conditions and efficacious assessment of physical rehabilitation exercise.

In particular, head pose estimation (HPE) is an important part of the whole evaluation process, which is the task of obtaining a 3D head pose deflection angle parameter through the analysis of a 2D digital image. The methods of head pose estimation can be broadly classified into two types based on whether they depend on landmarks.

**Landmark-dependent methods**: The core idea of this type of methods is to extract the landmarks on a human face first and then substitute these landmarks into the formula to calculate the head pose. In recent years, with the rapid development of convolutional neural networks (CNN), many CNN-based face landmark extraction methods have been proposed. For example, Liu et al. proposed a head pose estimation approach including two phases: the 3D face reconstruction and the 3D–2D matching keypoints. They first used CNNs, jointly optimized by an asymmetric Euclidean loss and a keypoint loss, to reconstruct a personalized 3D face model from the 2D image, and then used an iterative optimization algorithm to match the landmarks on the 2D image and the 3D face model reconstructed before under the constraint of perspective transformation [8]. Ranjan, R. et al. used a separate CNN to fuse the intermediate layers of a deep CNN and used a multi-task learning algorithm to operate on the fused features. The method was called HyperFace, performing the multiple tasks of face detection, landmarks localization, pose estimation and gender recognition at the same time, and outperformed many other competitive algorithms in each task [9]. Guo et al. proposed a regression framework called 3DDFA-V2, which utilizes a meta-joint optimization strategy to enhance speed and guarantee accuracy simultaneously [10]. However, CNNs often have difficulty solving the problem when the image structure does not conform to translation invariance. The graph convolutional neural network (GCN), on the other hand, can sometimes provide better results when dealing with graph structures, such as topological graphs made by connecting feature points of faces. Xin viewed head pose estimation as a graph regression problem. He built a landmark-connection graph and designed a novel GCN architecture to stimulate a complex nonlinear mapping relationship between the angle parameters of head pose and the graph typologies and overcame the unstable landmark detection with a joint Edge-Vertex Attention (EVA) mechanism [11]. Some methods regard head pose estimation as an auxiliary task, but also achieve good performance. Kumar, A. et al. proposed KEPLER to solve the unconstrained face alignment problem, and it also provides estimation of the head pose in three dimensions as a by-product [12].

**Landmark-independent methods**: This type of method has the advantage that the task of head pose estimation does not rely on the quality of the landmarks, which enhances robustness in a sense. Ruiz, N. et al. designed a loss including a pose bin classification and a regression component for each head pose Euler angle to compose a multi-loss network. When combined with data augmentation, their method has a good performance in estimating head poses in low-resolution images [13]. Huang et al. presented an estimation method using two-stage ensembles with average top-k regression to address the fragility of regression-based methods in estimating extreme poses [14]. Cao, Z. et al. used three vectors in a 9D rotation matrix to estimate head pose and adopted the mean absolute error of vectors (MAEV) to assess the performance [15]. Dhingra, N. proposed a light network LwPosr based on fine-grained regression using DSCs and transformer encoders, which can predict head poses efficiently with a very small number of parameters and memory [16]. Thai, C. et al. trained teacher models on the synthesis dataset to get pseudo labels of head poses. Then, they used a network based on ResNet18 to perform knowledge distillation and train the head pose estimation model with the ensemble of the pseudo labels [17].

Based on what has been mentioned above, landmark-independent methods perform slightly better than landmark-dependent methods in terms of accuracy of head pose estimation, but the network structures of landmark-independent methods are usually so heavy and complex that they are unsuitable for running on mobile and embedded platforms. In contrast, landmark-dependent methods run faster and have a smaller computational scale, which is more in line with the requirements for a lightweight estimation system in this task.

Head pose estimation has a wide range of applications in research areas such as driver attention detection [18,19], gaze estimation [20], iris recognition [21], face frontalization [22], best frame selection [23], dangerous behavior detection [24], and so on. However, although HPE has wide application prospects in the intersection of medicine and artificial intelligence, there are still few studies on the application of head pose estimation in the medical field, especially in the field of cervical spine health condition diagnosis and rehabilitation training guidance.

**Contributions**: To fill the vacancy in the area of an unmanned evaluating and monitoring system for cervical spine rehabilitation training, in this paper, we proposed a complete cervical spine health condition assessment system with a face encryption algorithm, and constructed a cervical exercise dataset which can be utilized in related studies. We adopted Mediapipe to acquire the necessary landmarks on human faces for the calculation of Euler angles. Mediapipe Face Mesh offers high-precision real-time detection of 468 3D face landmarks. Its lightweight architecture, together with GPU acceleration, enables the model to run on mobile devices with only a single camera input [25]. We estimated head movements based on certain landmarks and developed a correlation model between the head pose estimation algorithm and cervical spine rehabilitation. Moreover, we proposed a face encryption algorithm with controllable grain size to solve the privacy problem of patients in rehabilitation training.

The remaining chapters of this paper are as follows: Section 2 reviews related work and mentions some drawbacks of current methods. Section 3 summarizes the whole architecture of the evaluation method and analyzes each component in detail. Section 4 tests the repeatability of the head pose estimation algorithm, introduces the construction of the cervical spine operation dataset, analyzes the scoring results, performs an ablation study, and discusses the mosaic encryption effect. Finally, there is a conclusion summarizing the overall estimation process and presenting an outlook on future research directions.

## 2. Related Work

With the gradual maturation of computer vision technology, the application of computer vision in the medical field to improve the efficiency and accuracy of diagnosis has become a hot research topic in the academic community. However, little work has been done on the intersection of cervical spine health assessment and its rehabilitation guidance with computer vision, and the task focus and methods used in these works are different. For example, Xiong, J. et al. used computer vision and AR technology for acupuncture point selection in acupuncture therapy for cervical spondylosis and achieved an effective rate of 95.13% [26]. Murugaviswanathan, R. et al. proposed a method to automatically select points and calculate curvature for lateral views of the spine in videos using OpenCV [27]. This method can be transferred into the estimation of cervical vertebra. However, since in the actual scene, both the spine and the cervical vertebrae are wrapped with skin and muscle, rather than a thin curve exposed as in the text, this can cause great interference to this method of point selection and evaluation, making the results inaccurate. Jiang, J. addressed this problem by extracting the left edge after image enhancement of X-ray images of the cervical spine and analyzing the patient’s cervical spine health [28]. Wang, Y. et al. adopted an improved Yolov3 algorithm to extract the cervical vertebrae in infrared thermal images [29]. These two methods mentioned above have improved the precision of cervical spine extraction; however, these methods can only work after the patient’s cervical spine condition has been dissected using other cumbersome techniques (such as X-ray and thermal imaging), which is inconvenient and complicated. They can only process a single static image at one time and do not take into account a full range of factors when assessing the health of a patient’s cervical spine. These problems can all be solved in our method.

## 3. Methodologies

### 3.1. Model Architecture and Design

**Figure 1:**
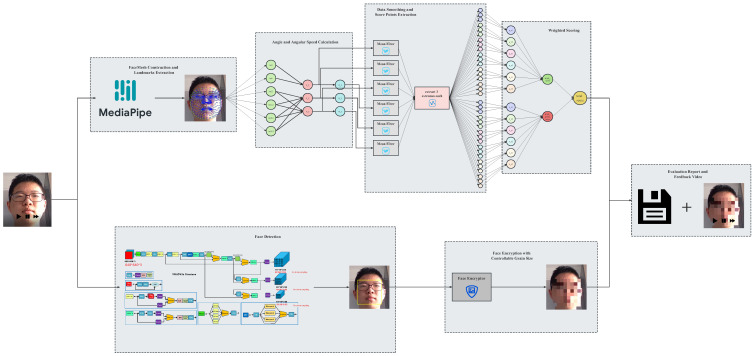
Model architecture.

The proposed evaluation model can calculate the head posture and other information by tracking the key points of the face in the input cervical spine exercise video and calculate the cervical spine score accordingly to generate a cervical spine health status assessment report; at the same time, it generates an assessment video with mosaic-processed face and real-time head posture data attached, which aims to provide scientific spine health status assessment and rehabilitation guidance while protecting the privacy of patients. The model architecture is shown below in Figure 1.

The specific working steps are as follows:

**Step 1: Face Landmarks Extraction**

The landmark points on the human face are first extracted using Mediapipe Face Mesh and are then inputted into the angle calculation module.

**Step 2: Euler Angle Calculation**

The formula that provides an accurate angle estimate with the least number of landmarks, is easy to understand, and reduces the computational overhead, is the one we choose for calculating the head pose based on coordinates.

**Step 3: Face Encryption**

YOLOv5 is used to detect and localize human faces, and grain-size-adjustable mosaics are put on the detected area to protect patients’ privacy.

**Step 4: Feedback Video Synthesis and Evaluation Report Generation**

Continuous head pose information is inputted in temporal sequence into the scoring system to get the final evaluation report, and a feedback video with encrypted face and head pose data attached is generated.

### 3.2. Scenario-Driven Improved YOLO Algorithm

You Only Look Once (YOLO) is a widely used algorithm, especially in the field of target detection. As its name suggests, the most important feature of YOLO is that it can output detection results at once after inputting the images to be predicted into the network, truly realizing the task of end-to-end target detection. Its lightweight network, extremely fast detection speed and excellent detection results make it widely used in many target detection-related scenarios. However, YOLO was not perfect in the beginning and many endeavors have been made by numerous scholars to improve YOLO’s performance.

The first version of YOLO is based on GoogLeNet, including 24 convolution layers and two fully connected layers [30]. It can perceive global information well while excluding the interference of background information, but there are still some problems, such as difficulty in detecting overlapping objects, inability to solve the multi-label problem, difficulty in detecting small objects, single aspect ratio of preselected boxes, etc. YOLOv2 uses Darknet-19 instead of GoogLeNet, adds batch normalization layers after all convolutional layers, adds 10 epochs of high-resolution fine-tuning during the training process, uses multi-label prediction, uses a K-means clustering algorithm to improve the size selection of anchor boxes, and adds a passthrough layer to increase detection accuracy [31]. YOLOv3 extends Darknet-19 to Darknet-53, using a residual network-like structure, and adopts multi-scale training. Another highlight in YOLOv3 is the strategy of ignoring the bounding boxes whose values of IOU are greater than a certain threshold, which is beneficial for improving the accuracy of predictions [32]. YOLOv4 replaces Darknet-53 with CSPDarknet-53, changes the activation function from ReLU to Mish, uses Dropblock to prevent overfitting, uses Mosaic data enhancement, cmBN, SAT self-adversarial training and other improvements on the input side, introduces an SSP module and FPN+PAN structure in the neck part, and changes IOU in the loss function to CIOU [33]. The biggest differences between YOLOX and the previous YOLO models are the application of the decoupled head, the adjustment in the process of data augmentation, and the deletion of anchor boxes (anchor-free) [34]. Recently, YOLOv6 has been proposed by Meituan’s team. It also uses the anchor-free approach and changes the backbone to EfficientRep, achieving great improvements in both detection accuracy and speed, especially with its impressive performance in small object detection in dense environments [35]. However, YOLOv6 is weaker in stability and less customizable in deployment compared with YOLOv5.

In the construction of our face encryption model, since the location and contour of the face in the video to be processed by the model are very clear and moderate in size, it is not necessary to pursue the ultra-high performance of the algorithm in the field of small target detection or other specific fields, but rather to focus on achieving a good balance between accuracy, processing speed, stability, flexibility, and ease of deployment. Taking the above factors into account, we decided that YOLOv5, with its high accuracy, fast detection speed, and customizable model, is the most suitable algorithm for our task.

YOLOv5 has four different networks, and the width and depth of the network can be adjusted according to the parameter configuration in the .yaml file [36]. On the input side, data augmentation, such as random mix-up, random scaling, and random cropping (similar to YOLOv4), is adopted for enhancing the generalization of the model; the process of calculating the initial value of the anchor box is changed to be embedded in the training code, and the method of pixel complementation of non-standard-size images is improved to reduce redundant information and improve the inference speed. In the backbone, the Focus structure is added, and the network structure of CSPDarknet + SPP is retained and improved. In the neck part, the structure of FPN + PAN is retained, and some ordinary convolutional layers in YOLOv4 are replaced by the CSP2 structure to strengthen the ability of network feature fusion. As is shown in Equation (1) (proposed in [37,38]), the loss function is CIoU_Loss, which takes into account the overlap area, aspect ratio, and centroid distance, avoiding the defects of IoU_Loss that cannot distinguish two boxes with the same intersection area but different intersection ways and gradient disappearance in the non-overlapping state.

(1) $$\left\{\begin{array}{cc} \; & IoU=\frac{|B_{gt}\cap B|}{|B_{gt}\cup B|}\\ \; & IoU\_loss=1-IoU\\ \; & v=\frac{4}{\pi^{2}}{\left(arctan\frac{w_{gt}}{h_{gt}}-arctan\frac{w}{h}\right)}^{2}\\ \; & \alpha =\frac{v}{(1-IoU)+v}\\ \; & CIoU\_loss=1-IoU+\frac{d_{center}^{2}}{d_{diagonal}^{2}}+\alpha v \end{array}\right.$$

Many other tricks are also used, such as abandoning the more complex activation function Mish and replacing it with LeakyReLU and Sigmoid to speed up inference; adopting a warm-up strategy when adjusting the learning rate to avoid using a larger learning rate at the beginning of training, which helps avoid early overfitting and ensure the stability of the model; and, as shown in Equation (2) (proposed in [38]), changing IoU_nms to DIoU_nms and adopting a weighting approach, which is conducive to more accurate detection of obscured or overlapping targets.

(2) $$\left\{\begin{array}{cc} \; & R_{DIoU}=\frac{d_{center}^{2}}{d_{diagonal}^{2}}\\ \; & s_{i}=\left\{\begin{array}{c} s_{i},IoU-R_{DIoU}<\varepsilon \\ 0,IoU-R_{DIoU}\leq \varepsilon \end{array}\right. \end{array}\right.$$

YOLOv5 is slightly weaker than YOLOv4 in terms of accuracy, but much stronger in terms of flexibility and prediction speed, and has a huge advantage in the rapid deployment of models.

### 3.3. Head Movement Estimation Algorithm

**Figure 2:**
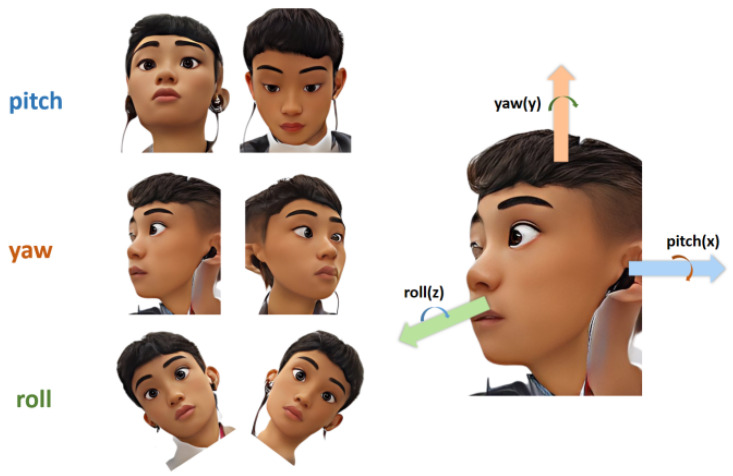
Three degrees of freedom to describe the movement of the head.

As is shown in Figure 2, three degrees of freedom can be used to describe the movement of the head: pitch, yaw, and roll.

Pitch represents the head-up and head-down movements, and the main muscles used are longus colli, longus capitis, infrahyoids, splenius capitis, seminispinalis capitis, suboccipitals, and the trapezius muscles. Yaw represents the movement of turning the head to the left or to the right, and the main muscles used are the levator scapula and suboccipitals. Roll represents the movement of tilting the head to one side so that the ear on that side gets closer to the shoulder on that side, and the main muscles used are the scalene muscles [39].

In this paper, feature extraction is performed based on the Mediapipe face detection algorithm and calculates the angles of head poses in three degrees of freedom. The formulas are shown below:

(3) $$\left\{\begin{array}{cc} \; & pitch:\alpha_{x}=arctan\left(k_{x}\right)\\ \; & yaw:\alpha_{y}=arctan\left(k_{y}\right)\\ \; & roll:\alpha_{z}=arctan\left(k_{z}\right) \end{array}\right.$$

where $k_{x}$, $k_{y}$, and $k_{z}$ is (lm is short for landmark)

(4) $$\left\{\begin{array}{cc} \; & k_{x}=\frac{z_{top\_nose}-z_{bottom\_nose}}{y_{top\_nose}-y_{bottom\_nose}}=\frac{z_{lm_{6}}-z_{lm_{2}}}{y_{lm_{6}}-y_{lm_{2}}}\\ \; & k_{y}=\frac{z_{right\_eye}-z_{left\_eye}}{x_{right\_eye}-x_{left\_eye}}=\frac{\frac{z_{lm_{263}}+z_{lm_{362}}}{2}-\frac{z_{lm_{33}}+z_{lm_{133}}}{2}}{\frac{x_{lm_{263}}+x_{lm_{362}}}{2}-\frac{x_{lm_{33}}+x_{lm_{133}}}{2}}\\ \; & k_{z}=\frac{y_{right\_eye}-y_{left\_eye}}{x_{right\_eye}-x_{left\_eye}}=\frac{\frac{y_{lm_{263}}+y_{lm_{362}}}{2}-\frac{y_{lm_{33}}+y_{lm_{133}}}{2}}{\frac{x_{lm_{263}}+x_{lm_{362}}}{2}-\frac{x_{lm_{33}}+x_{lm_{133}}}{2}} \end{array}\right.$$

**Figure 3:**
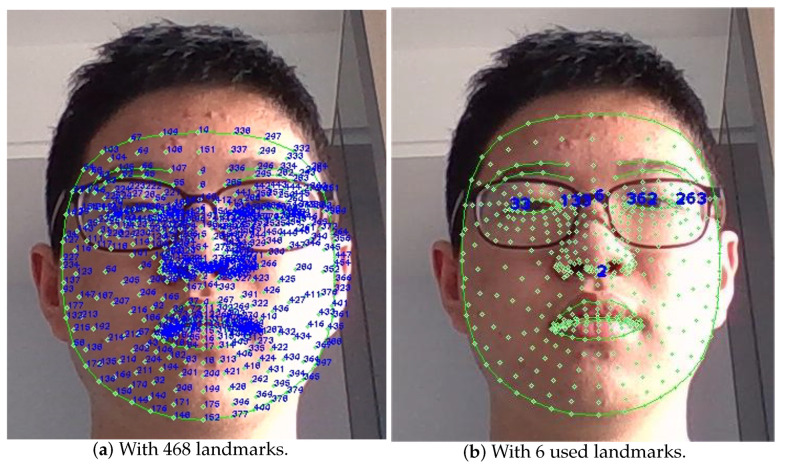
The face mesh landmarks.

The formulas in Equations (3) and (4) appeared in [40], although the expression of $k_{x}$ was incorrect. In this paper, the error is corrected and these formulas are further derived based on the selection of specific face feature points in our experiments. In this algorithm, as is shown in Figure 3, the face mesh with 468 landmarks in Mediapipe is used [41]. Six important landmarks are chosen to calculate angles in three dimensions, and the indices of these landmarks are as follows: 2 (bottom point of the nose), 6 (top point of the nose), 33 (left corner of the left eye), 133 (right corner of the left eye), 263 (right corner of the right eye), and 362 (left corner of the right eye). We can take the midpoint of the left corner and the right corner as the coordinates of the eye, and then, the tangent value of three angles can be calculated according to the coordinates of the left eye, the right eye, and two points on the nose.

### 3.4. Face Encryption Model

The mosaic is performed by dividing the selected face area into several small squares, each of which is unified into the color of the pixel in the upper left corner of the small square. The process of mosaicking is irreversible due to the loss of information during the encryption process, which effectively protects the patient’s privacy. Equation (5) for the pixel points at each location in the face region after being mosaicked is as follows.

(5) $$\left\{\begin{array}{cc} \; & x^{\prime }=x_{left}+\left\lfloor \frac{x-x_{left}}{mosaic\_size}\right\rfloor \times mosaic\_size\\ \; & y^{\prime }=y_{top}+\left\lfloor \frac{x-x_{top}}{mosaic\_size}\right\rfloor \times mosaic\_size\\ \; & color\left(x,y\right)=color\left(x^{\prime },y^{\prime }\right) \end{array}\right.$$

## 4. Experiment

### 4.1. Construction of Cervical Spine Operation Dataset

A complete set of cervical spine exercise movements was designed, including axial rotation, lateral bending, flexion, and extension in each direction three times.

To evaluate the method on videos of cervical vertebra rehabilitation, continuous videos were collected for two steps.

First, the cervical spine exercise of a volunteer was recorded as a standard video to test the repeatability of this algorithm. Then, another ten volunteers (5 males and 5 females with an average age of 16) were invited to do this exercise and the validation dataset for the scoring system was constructed.

**Figure 4:**
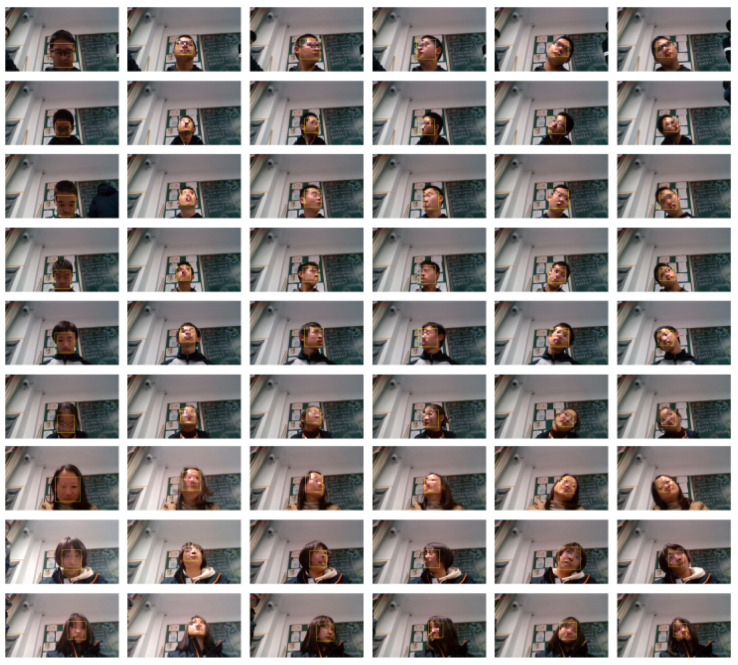
Different angles of the mosaic-processed faces.

All the volunteers’ movements were recorded by the OV9734 azurewave camera on a laptop at 30 fps, and Figure 4 shows six movements of some volunteers.

### 4.2. Analysis of Standard Cervical Spine Exercises

**Figure 5:**
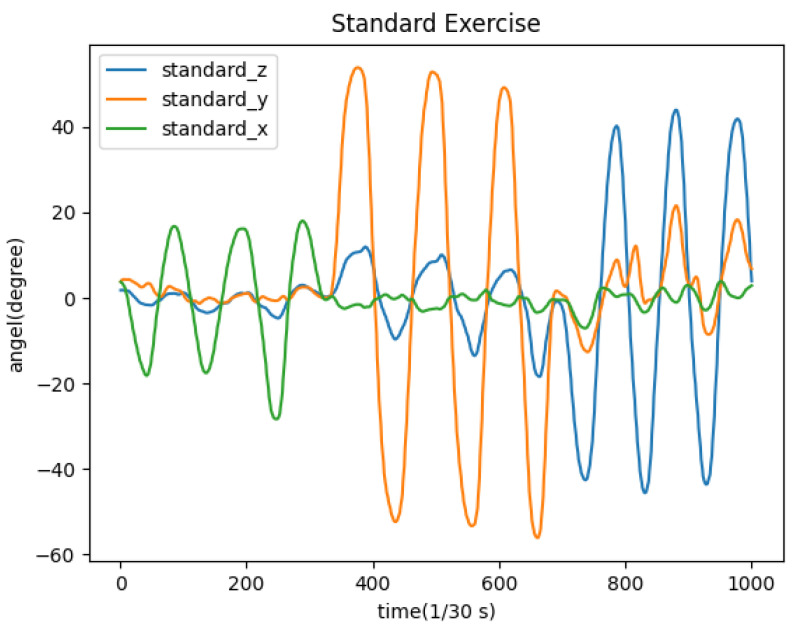
Changes of angles in the standard video.

Assuming the cervical spine exercise in one of the videos represents standard movements, the relationship between the standard angle and time is shown in Figure 5.

The same video is tested 10 times to evaluate the repeatability of the algorithm and the consistency of the angles calculated in three directions. We chose the standard deviation of the values of angles in each frame in 10 experiments as the measure of the repeatability of the algorithm. In this specific case, *n* = 10 in Equation (6).

(6) $$\begin{array}{cc} \; & s\left(x\right)=\sqrt{\frac{\sum_{i=1}^{n}{\left(x_{i}-\bar{x}\right)}^{2}}{n-1}} \end{array}$$

**Figure 6:**
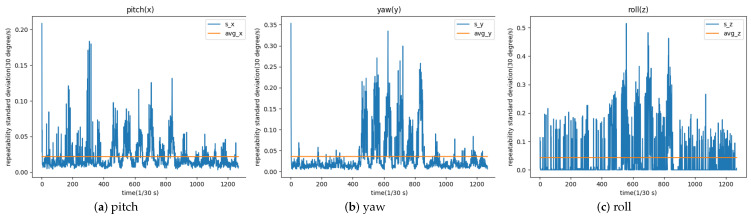
Standard deviation of angles in three directions.

The results are as shown in Figure 6.

Consistency test statistics charts are generated in three dimensions. The horizontal coordinate in each graph is the number of video frames, and the vertical coordinate is the standard deviation of the deflection angle of each frame in the video in one dimension after ten tests. The orange horizontal line is the average of the standard deviation of all frames, which can reflect the repeatability of our head pose estimation algorithm in that dimension. The average of the standard deviation in pitch, yaw, and roll is 0.0217, 0.0362, and 0.0432, which are all relatively low, and that means the algorithm has good repeatability.

## 5. Results and Discussion

In this section, a cervical spine condition scoring system is given and 10 volunteers (five males and five females) were tested and analyzed for their cervical spine condition using this system.

### 5.1. Scoring System

The range and speed of the head motion are two significant scoring indices of the health condition of the cervical spine. Calculation of head motion amplitude can detect problems such as joint misalignment and abnormal cervical curvature, while calculation of head motion speed can reflect the flexibility of the cervical spine and the degree of stiffness of related muscle groups. Many methods of assessing cervical spine health conditions only consider the range of head motion in all directions [42,43,44], that is, only the stationary state in which the patient performs an action is considered. This type of assessment, which is valid most of the time, sometimes misses some information. For example, a person with cervical spondylosis may be able to extend his neck slowly to the same angle as a healthy person, but this does not indicate that his cervical spine is as healthy as a healthy person’s cervical spine. If he stretches quickly, he may still feel pain, whereas a healthy person would not. Therefore, it is necessary to include the speed of head movements in the cervical spine health assessment system to evaluate the condition of the patient’s cervical spine under dynamic movement. Thus, the following scoring formula (7) is proposed:

(7) $$\begin{array}{c} score=\sum_{k\in \{x,y,z\}}\sum_{j=1}^{2}\sum_{i=1}^{3}\left(\frac{\min\{\alpha_{kj0},\alpha_{kji}\}}{\alpha_{kj0}}\times a_{kj}+\frac{\min\{\omega_{kj0},\omega_{kji}\}}{\omega_{kj0}}\times b_{kj}\right) \end{array}$$

where $\alpha_{x10},\alpha_{x20}$ means the standard head motion amplitude when doing flexion and extension, $\alpha_{y10},\alpha_{y20}$ means the standard head motion amplitude when doing axial rotation in left and right directions, and $\alpha_{z10},\alpha_{z20}$ means the standard head motion amplitude when performing lateral bending in left and right directions; $\alpha_{kj1},\alpha_{kj2},\alpha_{kj3}$ means the maximum three deflection angles in a given direction; ω means the angular velocity of the head movement and the meaning of its subscript is similar to that of α. We set 18 score points (6 directions × 3 extremes) for α and ω, respectively, and each score point is set as a full score if its value is greater than or equal to the standard value; if it is less than the standard value, it is assigned a score in proportion. The total full score is 100. Weighting factors $a_{kj},b_{kj}$ for each score point are displayed in Table 1.

Ohberg, F. et al. evaluated the importance of the range and the speed of head motion in [45], which is also the basis for the weighting of each score point in the above scoring formula.

### 5.2. Experiment Results

According to the research of Lind, B. et al. [46], the normal range of motion of the cervical spine is shown in Table 2.

**Figure 7:**
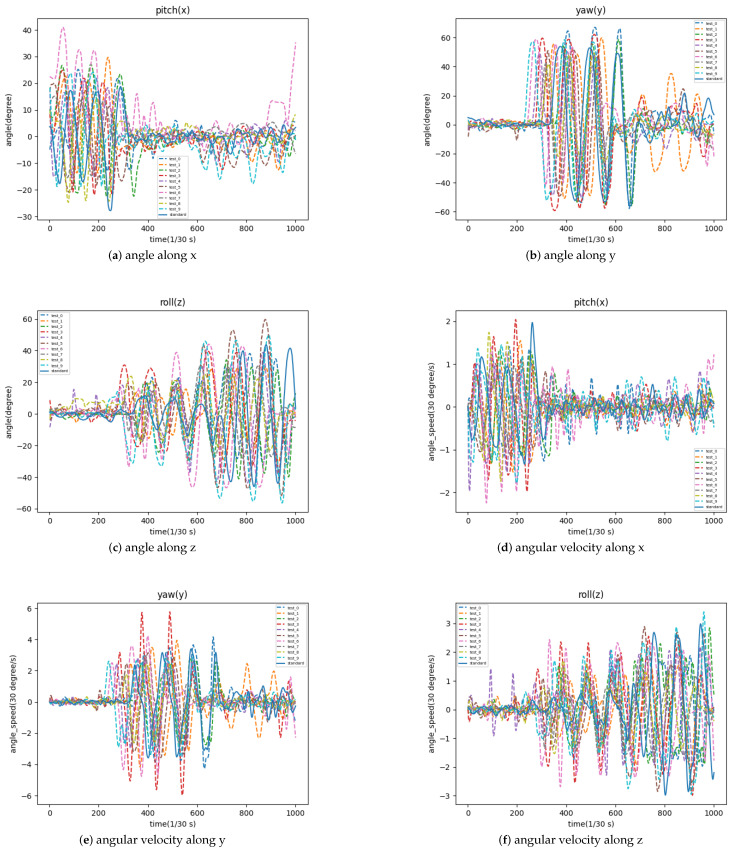
The variation of head deflection amplitude and angular velocity in three dimensions.

Additionally, a video of a volunteer’s cervical spine exercise was recorded as standard movements and the extreme values of angular velocity were calculated as standard values. The cervical exercise videos of 10 volunteers were tested with this scoring system and the variation of head deflection amplitude and angular velocity in three dimensions over the time domain are plotted in Figure 7.

### 5.3. Analysis of Experiment Results

**Figure 8:**
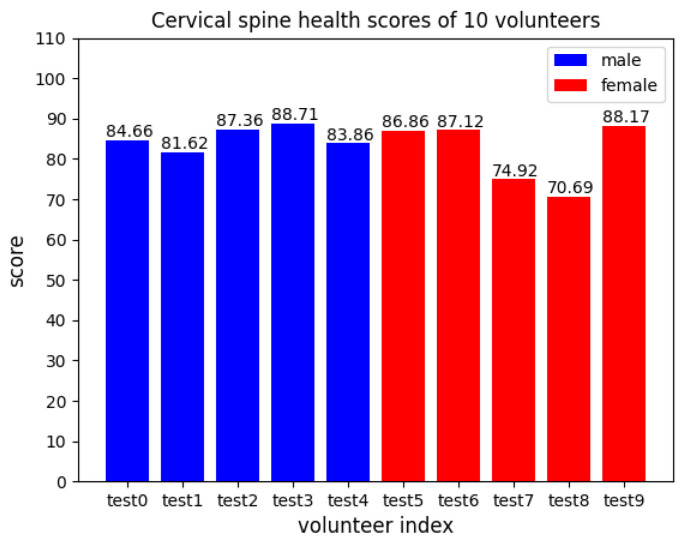
Cervical spine health scores of 10 volunteers.

The cervical spine health scores of 10 volunteers in Figure 8 are calculated according to the experiment results using the scoring system mentioned above.

**Figure 9:**
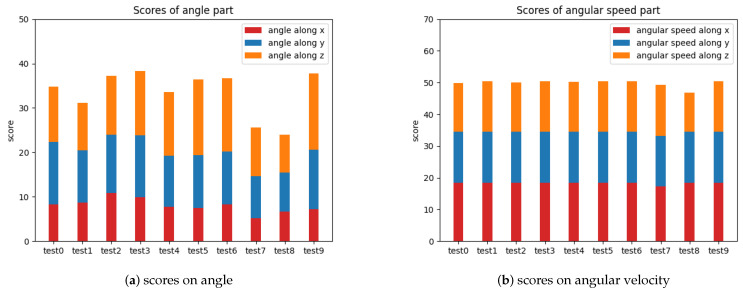
Detailed scores on head movement amplitude and movement angular velocity.

Their more detailed scores on head movement amplitude and movement angular velocity in the three dimensions are shown in Figure 9 below.

In a practical application scenario, the general health status of a patient’s cervical spine can be assessed based on the overall score, and the patient’s cervical spine problems can be localized based on the detailed score of each index.

### 5.4. Ablation Study

A human-in-the-loop method was adopted to evaluate and compare the quality of different scoring standards. Professional cervical spine physicians were invited to score the cervical exercise of each volunteer based on their medical examination reports and extensive clinical experience as physicians, and this assessment was set as the benchmark score. RMSE (root mean square error), MAE (mean absolute error), and $R^{2}$ (coefficient of determination) were calculated in different scoring standards. However, the human assessment of the absolute score of the quality of cervical spine exercises performed by a single patient is not always accurate, but the error is much smaller for the relatively good or bad assessment of the quality of cervical spine exercises performed by two patients. Considering the possible subjective errors in the human-in-the-loop method, KT (Kendall Tau) and RBO (rank biased overlap) are also calculated to assess the relative quality of cervical exercises performed by different patients, in order to assist in evaluating the quality of different scoring standards. The results are shown in Table 3. We can see that our method outperforms other scoring approaches in all evaluation metrics.

### 5.5. Discussion of the Mosaic Encryption Effect

**Figure 10:**
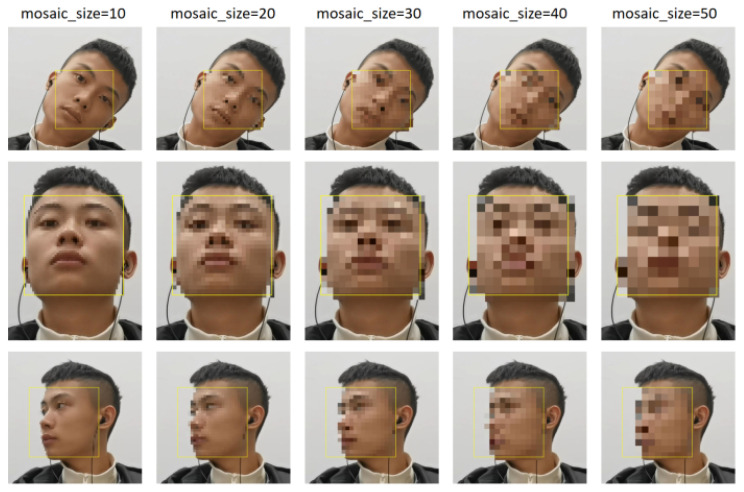
The encryption effect of five different sizes of mosaic on the face.

In the final output video, the size of the mosaic can be adjusted according to the resolution of the video, the diagnostic needs, the patient’s request for personal privacy, and other factors. The encryption effect of five different sizes of mosaic on the face was tested on our cervical exercise video with a resolution of 1920 × 1080, as shown in Figure 10.

## 6. Conclusions

In this paper, we proposed an evaluation method for cervical vertebral rehabilitation based on computer vision. The method first uses Mediapipe to construct a face mesh and extract several important landmarks to calculate the angles of head poses in three degrees of freedom. Then, the angular velocity in three dimensions over the time domain is calculated based on the angle data acquired before. After that, the health condition of the patient’s cervical vertebra can be evaluated generally and in detail using the scoring system. Finally, we use YOLOv5 to localize the human face, put mosaics on the detected area, and compose the full video with head posture information attached.

We validated the repeatability, scoring performance, and encryption effect of the proposed evaluation method by experiments based on the cervical exercise videos of volunteers with even gender distribution. The experimental results showed that our algorithm has good repeatability and that our scoring system can effectively reflect the health status of the patient’s cervical spine.

In the future, we plan to work with hospitals to access cervical spine data from real patients to expand the dataset to patients all of ages and study the correlation between changes in various indicators of the patient’s cervical spine during treatment and the patient’s recovery progress, and further improve the assessment indicators and weights in the scoring system. In addition, we will also try to expand and transfer this method of assessing patients’ rehabilitation status with the help of machine vision to more medical fields to better utilize the advantages of cross-disciplinary research.

## Figures and Tables

**Table 1:** Weighting factors for each score point.

	x1	x2	y1	y2	z1	z2
a	2.25	2.82	2.90	2.82	2.90	2.82
b	3.06	3.09	2.75	2.58	2.75	2.58

**Table 2:** Standard value of angles in three directions.

	Pitch/x (Degree)	Yaw/y (Degree)	Roll/z (Degree)
Male	68	145	45
Female	76	139	45

**Table 3:** Comparisons between different scoring standards.

	RMSE	MAE	R${}^{2}$	KT	RBO
angle_all	16.353	15.879	−6.220	**0.9111**	**0.975**
angular_speed_all	15.797	15.192	−5.737	0.645	0.853
angle_x + angular_speed_x	7.307	6.280	−0.442	0.2889	0.708
angle_y + angular_speed_y	2.964	2.492	0.763	0.644	0.892
angle_z + angular_speed_z	7.328	5.917	−0.450	0.689	0.862
angle_xy + angular_speed_xy	4.214	3.325	0.521	0.511	0.733
angle_xz + angular_speed_xz	1.649	1.356	0.927	0.733	0.908
angle_yz + angular_speed_yz	3.150	2.672	0.732	0.867	0.957
angle_all + angular_speed_all (Ours)	**0.765**	**0.586**	**0.984**	**0.9111**	**0.975**

## Data Availability

The data for this article can be obtained by contacting the authors by email.
